# Supplementary material for: Spatial Variation in Stream-Water Bacterial Communities Across Sites Differing in Wild Chinese Giant Salamander Detection Frequency
Source: Biology (Basel). 2026 Sep 18;15(18):1647. doi: 10.3390/biology15181647 (PMC13604818; doi:10.3390/biology15181647)
Supplement: Supplementary file 1 [file biology-15-01647-s001.zip › Data Related to “Spatial Variation in Stream-Water Bacterial Communities across Sites Differing in Wild Chinese Giant Salamander Detection Frequency”/Microbial Data/report/src/hyperlinks/QC_methods_LRS_EN.pdf]

Method

The method is only used as a reference for publication purpose. Customers are responsible for the related risks of duplicate checking.

Experimental Procedure

Genomic DNA Extraction and Quality Assessment

Genomic DNA was extracted according to sample type. The extracted DNA was then subjected to quality assessment via 1% agarose gel electrophoresis (120V, 30 min) to evaluate band integrity and nucleic acid purity.

Qualified DNA samples were diluted with sterile nuclease-free water to a final concentration of 1 ng/μL for downstream experiments.

PCR Amplification and Product Purification

The diluted genomic DNA was used as the template for PCR amplification of the target 16S rRNA gene region using barcode-tagged primers. PCR was performed in a reaction containing 12.5 μL KAPA HiFi HotStart ReadyMix, 2.5 μM primers, and 10 ng genomic DNA template. The thermal cycling conditions were as follows: initial denaturation at 95℃ for 3 min, followed by 22 cycles of denaturation at 98℃ for 20 s, annealing at 57℃ for 30 s, and extension at 72℃ for 75 s, with a final extension at 72℃ for 5 min. The PCR products were verified by 2% agarose gel electrophoresis and purified using magnetic bead-based purification kits (e.g., AMPure XP) to remove primer dimers.

Kinnex Library Preparation

Barcoded PCR products from different samples were pooled in equimolar amounts, and the Kinnex long-read sequencing technology was employed for library preparation. The detailed steps are as follows:

End Repair: PCR products were treated with Kinnex End Repair Enzyme (20°C, 30 min) to ensure blunt-ended DNA fragments.

Adapter Ligation: Kinnex-specific sequencing adapters (20 μM) and T4 DNA ligase (20°C, 15 min) were added to ligate DNA fragments to sequencing adapters.

Linear Array Assembly: The 16S rRNA gene fragments were concatenated into

long linear DNA arrays using Kinnex Assembly Enzyme (37°C, 1 h).

Library Purification: SMRTbell® magnetic beads were used for size selection (0.45× and 0.8× bead ratios) to retain target fragments ranging from 500 to 2000 bp, thereby improving library quality.

Library Quality Evaluation and Sequencing

The purified library was quantified using a Qubit® 4.0 Fluorometer, and its size distribution was assessed using an Agilent 2100 Bioanalyzer to ensure the primary peak fell within the expected range (1.5–3 kb). Libraries meeting quality standards were sequenced on the Revio platform using Single Molecule, Real-Time (SMRT) sequencing to obtain high-quality long-read data.
